# Diversity and Local Coadaptation of *Escherichia coli* and Coliphages From Small Ruminants

**DOI:** 10.3389/fmicb.2020.564522

**Published:** 2020-10-16

**Authors:** Felipe Molina, Alfredo Simancas, Rafael Tabla, Antonia Gómez, Isidro Roa, José Emilio Rebollo

**Affiliations:** ^1^Department of Biochemistry, Molecular Biology and Genetics, University of Extremadura, Badajoz, Spain; ^2^Dairy Department, Technological Institute of Food and Agriculture – Scientific and Technological Research Centre of Extremadura, Junta de Extremadura, Badajoz, Spain

**Keywords:** phage-host coadaptation, *Escherichia coli*, biocontrol, raw milk cheese, phage therapy, tradeoffs in life history, dairy, bacteriophages

## Abstract

Bacteriophages are highly specific predators that drive bacterial diversity through coevolution while striking tradeoffs among preserving host populations for long-term exploitation and increasing their virulence, structural stability, or host range. *Escherichia coli* and other coliform bacteria present in the microbiota of milk and during early ripening of raw milk cheeses have been linked to the production of gas, manifested by the appearance of eyes, and the development of off-flavors; thus, they might cause early blowing and cheese spoilage. Here, we report the characterization of coliphages isolated from manure from small ruminant farms and *E. coli* strains isolated from goat and sheep raw milk cheese. Additionally, the virulence and host range of locally isolated and laboratory collection phages were determined by comparing the susceptibility of *E. coli* strains from different sources. In agreement with the high genetic diversity found within the species *E. coli*, clustering analysis of whole-cell protein revealed a total of 13 distinct profiles but none of the raw milk cheese isolates showed inhibition of growth by reference or water-isolated coliphages. Conversely, 10 newly isolated phages had a broad host range (i.e., able to lyse ≥50% of bacterial hosts tested), thus exhibiting utility for biocontrol and only one cheese-isolated *E. coli* strain was resistant to all the phages. Whereas there was a high positive correlation between bacterial susceptibility range and lysis intensity, the phages virulence decreased as range increased until reaching a plateau. These results suggest local gene-for-gene coevolution between hosts and phages with selective tradeoffs for both resistance and competitive ability of the bacteria and host-range extension and virulence of the phage populations. Hence, different phage cocktail formulations might be required when devising long-term and short-term biocontrol strategies.

## Introduction

The mysterious demise of Lord Carnarvon after entering Tutankhamen’s tomb, although wrongly attributed to exposure to deadly mycotoxins (Cox, 2003), served to pose “the curse of the pharaoh theory,” which postulates that extreme structural stability should favor the evolution of high virulence in parasites (Gandon, 1998; Roche et al., 2011). However, in bacteriophages, “life” history tradeoffs arise due to environmental stressors, proteins governing viral host range, and organization of the compact genome (Goldhill and Turner, 2014). Hence, successful phages must strike a balance between maximizing their virulence and preserving host populations for long-term exploitation, implying that evolving reduced virulence avoids eradicating the bacterial population or driving it toward total resistance. Whereas some phages exhibit a broad host range (Balogh et al., 2010), infecting different genera, the diverse nature and location of the host cell receptors usually entails high specificity of phage-host interactions (Bertozzi Silva et al., 2016). Moreover, phage extracellular existence produces a compromise among rapidly and successfully infecting hosts, withstanding environmental stressors (such as extreme pH and temperature) and spurious adsorption to inappropriate targets (Gallet et al., 2009; Keen, 2014). Like their predators, subtle tradeoffs between resistance and competitive ability determine the composition of bacterial populations when phages are present and hinder phage-resistant lineages from completely overcoming sensitive bacteria and eradicating predating phages (Bohannan and Lenski, 2000). Thus, the cost of resistance mutations is determined by the extent of resistance conferred, interactions with the abiotic environment and genetic backgrounds (Bohannan and Lenski, 1999). Resistance mutations to phage attacks might produce an evolutionary tradeoff in multi-drug resistant bacteria, whereby the selection of resistance to phages changes the efflux pump mechanism, causing increased sensitivity to several antibiotic classes (Chan et al., 2016). Whereas partial resistance is less costly than complete resistance (Lenski, 1988), mutations that confer cross-resistance to unrelated coliphages tend to be the most costly (Sen and Nikaido, 1991). Additionally, the level of T4 resistance in *E. coli* varies with the carbon source (Bohannan and Lenski, 1999) and temperature (Bohannan and Lenski, 2000).

In addition to these tradeoffs, coevolution shapes the diversity of phage-host communities. Negative frequency-dependent selection elicits diversification of bacteria (to escape phages) and phages (to chase evolving bacteria). Hence, local adaptation creates modular networks of hosts and phages maintained by kill-the-winner ecological dynamics and a matching allele model (Flores et al., 2011). Accordingly, the cost of new mutations leading to infect/resist recently evolved hosts/phages would entail the loss of infectivity/resistance against ancestral genotypes (Agrawal and Lively, 2003). However, at small scales, phage–bacteria interaction networks typically show a nested structure, in which both hosts and phages can be ranked by their range of resistance and infectivity, respectively (Beckett and Williams, 2013). The nestedness derives from gene-for-gene processes, so host-range expansion among phages evolves without compromising the ability to infect ancestral host genotypes, and likewise, the appearance of new resistance mutations in bacteria maintains resistance to past phages (Weitz et al., 2013). Experimental quantification of phage-host coevolutionary dynamics (Fortuna et al., 2019) has revealed that the nestedness decreases over time under fluctuating dynamics but increases under arms race dynamics. Moreover, when host-range is broad, phages and bacteria diversify more under arms race dynamics than under fluctuating dynamics. Since host range is a key property of phage therapy, it seems important to keep these considerations in mind while looking for effective bacteriophages.

*Escherichia coli* and other coliform bacteria are indicators of unfavorable hygienic conditions and fecal contamination (Molina et al., 2015), and some strains may cause severe disease in mammals and economic losses for food producers (Abe et al., 2002; Grad et al., 2012). *E. coli* exhibits remarkable genomic variation, and only 6% of its gene families are represented in every genome, comprising the core genome (Lukjancenko et al., 2010). Additionally, the high genomic similarity with related species suggests blurred species borders between *Shigella* spp. and *E. coli* (Gordienko et al., 2013). As a consequence of the resulting phenotypic variation, *E. coli* plays numerous biological roles, from laboratory-adapted workhorses to beneficial commensal or intracellular pathogens (Hendrickson, 2009). A substantial amount of antagonistic pleiotropy in evolved populations, as well as metabolic tradeoffs, are commonly found (Azevedo et al., 2016). These divergent niche adaptations hamper the achievement of broad host range phages for biocontrol of *E. coli* lineages.

Although cheese made from raw milk usually has intense flavor because of its diverse and highly variable microbiota (Montel et al., 2014), *E. coli* might cause early blowing and cheese spoilage (O’Sullivan and Cotter, 2017). Thus, the presence of *E. coli* during early ripening of raw milk cheeses has been linked to the production of gas, manifested by the appearance of eyes, and the development of off-flavors (Guggisberg et al., 2015; Tabla et al., 2016, 2018). Raw milk cheeses, particularly soft and semihard cheeses, have been associated with pathogenic *E. coli* outbreaks (Altekruse et al., 1998). Conversely, high levels of non-pathogenic *E. coli* in raw milk cheeses may contribute to the development of desirable characteristics of some of these products (Zago et al., 2007). The main advantage of using phages as biocontrol agents against undesired bacteria in cheese is that the specificity of phage-host spare the microbiota responsible for infusing natural flavors from being killed during treatment (Pujato et al., 2018). Conversely, the narrow host range limits the use of a single-phage treatments and multiple phage species are often mixed into cocktails (Abedon et al., 2017).

Here, we report the characterization of coliphages isolated from ewe feces and *E. coli* strains isolated from goat and sheep raw milk cheese. Whole-cell protein bacterial profiles and host-range clusters were compared. The virulence and host range of locally isolated and laboratory collection coliphages were determined by comparing the phage susceptibility of diverse *E. coli* strains. Several phages had a broad host range (i.e., able to lyse ≥50% of bacterial hosts tested), thus exhibiting utility for *E. coli* biocontrol. Our results suggest local gene-for-gene coevolution between hosts and phages, with selective tradeoffs for both resistance and competitive ability of the bacteria and host-range extension and virulence of the coliphage populations.

## Materials and Methods

### *Escherichia coli* Strains and Bacteriophages Used as References in This Work

The bacterial strains and coliphages used as references in this work are listed in Table 1. *E. coli* K-12 MG1655 was used for phage propagation. All bacterial cultures were grown in Luria broth (Pronadisa, Spain) at 37°C for 18–24 h.

**TABLE 1:** Bacterial strains and bacteriophages used as references in this study.

Strain or phage	Source and references
***Escherichia coli***	
K12 (MG1655)	This lab (Molina et al., 1998) ATCC 700926
MG1655 λ +	This lab
B (Luria)	CECT 4201
B/r	CECT 105
Bi	CECT 4537
BW6164	CGSC 6759
C (Sinsheimer)	ATCC 13706
W1 (Waskman)	CECT 99
W2 (Stoke)	CECT 727
C600	Gifted by Dr. Rouviere-Yaniv ATCC 23724
GY752	This lab (Sommer et al., 1998)
VIP45 λ+	This lab (Dr. Miguel Vicente)
***Shigella***	
*Shigella boydii*	CECT 583
*Shigella flexneri* 2a	CECT 585
*Shigella flexneri* 2b	CECT 4804
*Shigella sonnei*	CECT 4887
**Bacteriophages**	
λ	This lab, VIP45 strain induction
P1*vir*	NIG HR16
T4	NIG HR17
T6	NIG HR18
SOM2, SOM3, SOM5, SOM8, SOM15	*Myoviridae* isolated from seawater (Muniesa et al., 2002)
SCH2, SCH10	*Myoviridae* isolated from sewage (Muniesa et al., 1999)
SCH5	*Siphoviridae* isolated from sewage (Muniesa et al., 1999)
STER5	*Myoviridae* isolated from river water (Muniesa et al., 1999)

*ATCC, American Type Culture Collection (United States); CECT, Colección Española de Cultivos Tipo (Spain); CGSC, Coli Genetic Stock Center (United States); NIG, National Institute of Genetics (Japan).*

### Cheese Manufacturing and Sample Taking

Two batches of soft ewe cheese (Torta del Casar PDO), semihard goat cheese (Ibores Cheese PDO) and semihard ewe cheese were manufactured (∼0.650 kg), each by two different local producers, following traditional methods. The soft variety was made according to the PDO regulation (Official Journal of the European Union [OJEU], 2002) with the addition of vegetable coagulant (*Cynara cardunculus*). Semihard varieties were made with the addition of calf rennet. “Ibores cheeses” were made according to the PDO regulation (Official Journal of the European Union [OJEU], 2004). Semihard ewe cheese was made by adding lyophilized direct-to-vat mesophilic mixed culture (R–704, 50 units; Chr. Hansen, Hørsholm, Denmark) containing *Lactococcus lactis* subsp. *cremoris* and *L. lactis* subsp. *lactis* as the starter culture. Cheeses were brine salted (16° B at 10°C for 6 h) and ripened at 10–12°C and 80% relative humidity for at least 60 days. From each of the batches, samples of milk, curd, and 1-, 2-, 4-, and 8-week-old cheese were taken and kept refrigerated for no longer than 8 h before analysis.

### Detection of Coliforms

Preparation of test samples for microbiological examination was performed according to ISO 6887-5:2010. Milk samples were diluted in 9 mL of 0.1% (w/v) peptone saline water at 30°C and subjected to serial dilutions. For curd and cheese, representative 10 g samples were placed into a sterile stomacher bag with 90 mL of sterile 2% (w/v) trisodium citrate solution (Panreac, Barcelona, Spain) and blended for 5 min at 40°C in a stomacher (Stomacher Type 400; Seward, London, United Kingdom). Serial dilutions were prepared in 0.1% (w/v) sterile peptone water and plated on *E. coli* coliform chromogenic medium (Pronadisa, Spain). Red to pink colonies were considered presumptive coliforms, and dark-blue to violet colonies were presumed *E. coli*.

### Isolation, Identification and Characterization of *Escherichia coli*

Up to 10 presumptive *E. coli* isolates, identified by positive β-D-glucuronidase (MUG discs, Remel, United States) and β-D-galactosidase (ONPG discs, Oxoid, United Kingdom) activities, were randomly taken from each count plate and isolated by three alternate subcultures in nutrient broth (Oxoid Ltd., Hampshire, United Kingdom). Gram–negative, oxidase–negative and catalase–positive isolates were stored in LB medium (Pronadisa, Spain) containing 30% (v/v) glycerol at −80°C.

Identification was performed with the aid of an EnteroPluri-Test System (Liofilchem^®^, Italy). Additionally, over 10% of the total number of isolates was analyzed with a Biolog Microbial ID System (Biolog, United States) according to the manufacturer’s instructions. Strain characterization was performed by one-dimensional sodium dodecyl sulfate-polyacrylamide gel electrophoresis (SDS-PAGE) of whole-cell proteins. Protein samples were prepared according to (Jackman, 1988) and analyzed by 10% SDS-PAGE by the method of (Laemmli, 1970). Gels were digitized using a densitometer (Bio-Rad GS800) and were analyzed with the aid of Phoretix 1D Pro software (Non-linear Dynamics, Newcastle, United Kingdom). The reference *E. coli* strain (ATCC 13706) was also included in the analysis. Protein patterns were corrected for gel distortion and gel–to–gel variation using a reference bacterial standard (*Hafnia alvei*, CECT 158). The similarity among digitized profiles was calculated using Pearson correlation, and an average linkage (UPGMA) dendrogram was derived from the similarity matrix.

### Isolation of Bacteriophages

A total of 16 sheep feces samples (25 g) were collected from four local ewe dairy farms (four samples per farm). Each sample was homogenized with 100 mL of phage suspension buffer [1% 1 M MgSO_4_ and 0.5 M CaCl_2_ (v/v)]. After 2 h of incubation at room temperature in a stomacher, samples were filtered and centrifuged at 8000 × *g* for 10 min. After adding a few drops of trichloromethane, the samples were incubated for 15 min at 37°C and centrifuged again at 8000 × *g* for 10 min. The supernatant was filtered through 0.22 μm pore diameter filters (MF; Millipore) and stored at 4°C in glass vials with trichloromethane to kill the remaining bacteria. To detect the presence of coliphages in the supernatants, *E. coli* k-12 MG1655 was seeded using the double-layer method. Plates were divided into sectors, and aliquots of 20 μl of phage supernatant were added to each sector. Once dried, plates were incubated at 37°C for 18 h to allow lytic zones appear. Phage strain isolation was initially carried out by spot assay as described elsewhere (Mirzaei and Nilsson, 2015). Later, to purify individual phages, the center of the plaques was pierced with a pipette tip dipped in 100 μL LB and diluted 1000-fold. Finally, 10 μL were mixed with 100 μL of a fresh bacterial culture (OD600 nm = 0.2–0.4), incubated 10–15 min before transferring to fresh 2.5 mL heated (50°C) soft agar (0.6%) and plated on LB agar. The latter step was repeated at least twice until all plaques were homogenous. A total of 88 distinctive plaque-forming units at this stage were treated as different phages.

### Determination of Plaque Size and Adsorption Rate

To measure plaque size, the phage strains were plated using *E. coli* strain MG1655 as the host strain and using double-layer agar as described elsewhere (Sambrook and Russell, 2001). After overnight incubation at 37°C, plates were analyzed with an automatic colony counter (Scan 500, Interscience) set to illuminate from below and detect dark plaques over clear backgrounds. Sensitivity and minimal detectable size thresholds were adjusted manually when needed. The mean plaque diameter was determined after measuring at least 100 plaques.

Lambda phage (Table 1) adsorption to host cells was performed as described previously (Gabig et al., 2002). Host strains were grown in LB (Pronadisa, Spain) to an OD600 nm of 0.1 and incubated with λ phage (multiplicity of infection: 0.1) in a water bath at 37°C with low constant shaking. At 5, 10, 15, and 30 min after the infection, aliquots were centrifuged at 3000 × *g* and 4°C for 5 min to allow attached phages to sediment along with bacteria while leaving free viral particles in the supernatant. The resulting samples were titrated for bacteriophages by triplicate plaque assays using six plates per assay. The decrease in the free phage titer normalized with respect to the initial phage titer indicates the adsorption rate. Theoretical phage adsorption kinetic data were taken from Storms and Sauvageau (2015).

### Cross-Streak and Host-Range Determination

Phages (10^7^ pfu/mL) were plated in nutrient agar (Oxoid Ltd., Hampshire, United Kingdom) following parallel streaks across the plate. Once dry, bacteria (10^8^ pfu/mL) were plated perpendicular to phage streaks. After overnight incubation at 37°C, a picture of each plate was digitized using a colony counter (Scan 500, Interscience). Zones of bacterial lysis were assessed with a scaling system, where 0 indicated no infection and 3 indicated a fully or nearly fully degraded bacterial lawn. Each infection assay was performed three times, and the average values were converted into a heat map.

### Transmission Electron Microscopy

Transmission electron microscopy (TEM) was performed as previously described (Casey et al., 2015). Negative staining was performed using 2% (w/v) uranyl acetate on freshly prepared ultrathin carbon films (Plano, Wetzlar, Germany). Grids were analyzed in a Tecnai G2 20 transmission electron microscope (FEI Thermo Fisher Scientific) at an acceleration voltage of 80 kV.

### Statistical Analysis

Two-tailed (95% confidence interval) non-parametric Spearman correlation tests were performed where indicated. To evaluate the correlation between whole-cell protein profiles and susceptibility range of raw milk cheese *E. coli* isolates, a Mantel test was carried out using distance matrices. To estimate the *p*-value, 9909 Monte Carlo simulations were carried out with a significance level of 5%, 10,000 permutations, 6953 seeds (random numbers) and a confidence interval of 0.000–0.002.

## Results and Discussion

### Characterization of *Escherichia coli* Isolated During Raw Milk Cheese Manufacturing and Ripening

**FIGURE 1:**
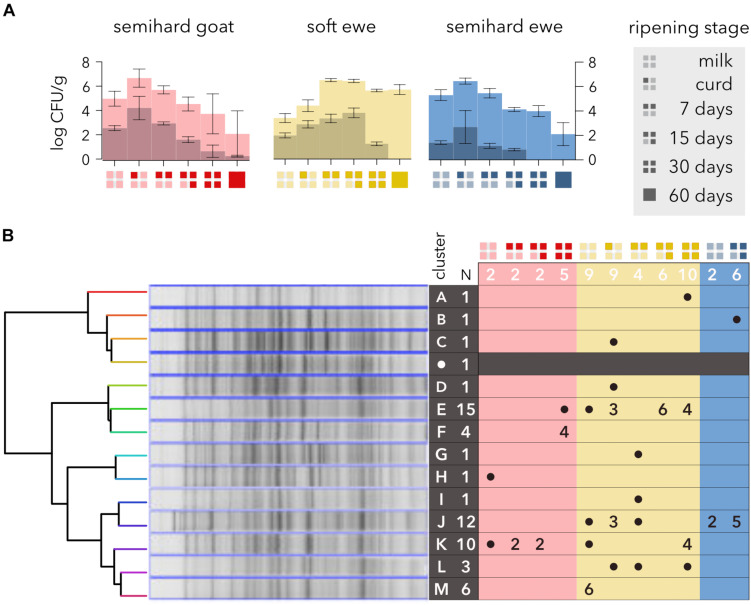
Evolution and whole-cell protein profiling of *Escherichia coli* during different manufacturing and ripening stages of raw milk cheeses. **(A)** Changes in *E*. *coli* concentration (dark shading) and in total coliform bacterial levels (light shading). The semihard goat, soft ewe, and semihard ewe cheese varieties are represented by red, yellow, and blue, respectively. The ripening stages are represented by the code shown in the legend. **(B)** Hierarchical classification of *E. coli* strains based on their SDS-PAGE protein profiles. Strains were clustered from a similarity matrix, and a representative SDS-PAGE profile from each cluster is shown. A reference *E. coli* strain (CECT4622) is included (white dot on dark row). The total number of strains in each cluster is shown in row headers. The number of isolates from each sample is shown in column headers.

*Escherichia coli* was isolated on chromogenic medium from every raw milk sample (Figure 1A) ranging from 1.4 to 2.6 log CFU/g, whereas coliforms were from 1.5 to 3.5 orders of magnitude more abundant. These *E. coli* levels are noticeably low compared with those from soft or semisoft cheeses made from raw cow milk (Zago et al., 2007). In semihard cheeses, the maximum counts were detected during the first week of ripening, but in soft ewe cheese, the maximum was achieved during the second week. The numbers of *E. coli* decreased during ripening until their eventual elimination at 60 days. Strikingly, coliform levels did not decline noticeably in soft ewe cheese. Semihard cheese is usually ripened at 10°C, acidifying the cheese during the first week, while soft cheeses ripen below 8°C, which slows down the acidification and might explain these differences. *E. coli* has been reported as the predominant species toward the end of the ripening period in several cheese varieties (Gaya et al., 1987; Tornadijo et al., 1993; Freitas et al., 1996), showing its resistance to the adverse conditions of ripened cheeses. The extinction of *E. coli* might be due to competence with the microbial consortium, probably with *H. alvei*, which has been related to *E. coli* inhibition (Delbès-Paus et al., 2013; Callon et al., 2016), and is the predominant *Enterobacteria* after 30 days of ripening in these cheese varieties (Tabla et al., 2016, 2018).

Two-thirds (57 out of 86) of β-D-glucuronidase- and β-D-galactosidase-positive colonies were confirmed as *E. coli* by two additional methods (data not shown) and selected for further characterization using one-dimensional SDS-PAGE of whole-cell proteins (Figure 1B). Although chromogenic media have been validated to identify total coliforms and *E. coli* in foods (Turner et al., 2000; González et al., 2003), disagreement between different methods for the identification of *E. coli* and coliforms has been widely reported. Hence, whereas a high proportion of β-D-glucuronidase-negative *E. coli* strains has been described (Chang et al., 1989; Feng et al., 1991), β-D-glucuronidase activity is found in other bacterial species (Frampton and Restaino, 1993; Molina et al., 2015). Some presumptive *E. coli* isolates from raw milk cheese were eventually ascribed to the genus *Klebsiella* (Zago et al., 2007).

Clustering analysis of PAGE total protein profiles based on UPGMA clustering and cosine coefficient revealed at the 90% similarity level a total of 13 distinct protein profiles (Figure 1B). These results are in agreement with the high phenotypic diversity found in strains isolated from raw cow milk cheeses (Zago et al., 2007) and with the high genetic diversity found within the species *E. coli* (Lukjancenko et al., 2010). Three types of proteomics profiles could be distinguished: seven clusters comprised only one isolate, three others (F, L, and M) comprised a few isolates (3–6) and the rest (E, J, and K) depicted higher numbers (10, 12, and 15). Only the latter were found in two cheese varieties, but none of the clusters was ubiquitous enough to be found in every cheese sampled. Two clusters (E and K) were found in goat and soft ewe cheese, one (J) and both ewe cheeses and none in goat and semihard ewe cheese. The numbers of clusters found at different ripening stages were similar, four clusters at week 2 and 5 at the other ripening stages and two clusters (H and M) were detected only in milk, probably due to sensitivity to acidity, which may cause their disappearance during cheesemaking and ripening.

### *Escherichia coli* Strains Isolated From Cheese Are Not Lysed by Reference or Water-Isolated Coliphages

**FIGURE 2:**
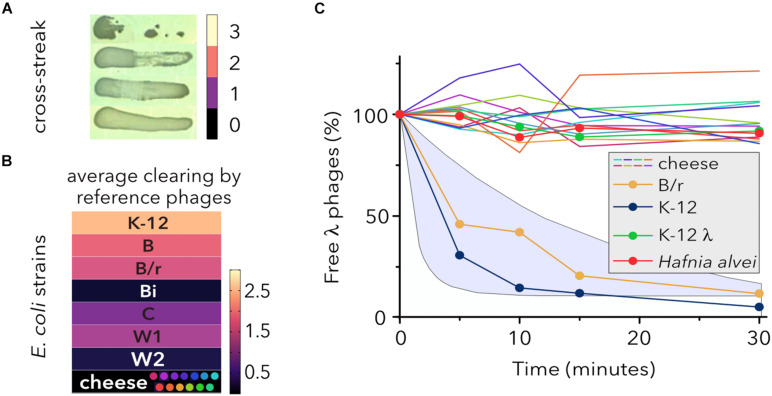
Susceptibility of *E. coli* strains to different coliphages. **(A)** Cross-streak analysis of bacterial lysis. Zones of clearing were assayed with a scaling system where 0 indicates no lysis and 3 indicates a completely clear zone. Representative examples are shown. **(B)** Susceptibility of *E. coli* lineages to reference phages (λ, T4, T6, and P1) and nine phage isolates from water sewage, rivers, and seawater (Table 1). One *E. coli* strain (data not shown) of each type was tested. The colored dots represent the SDS-PAGE protein profiles of raw milk-isolated strains. The average clearing values, after single infections with reference phages and two assays for each phage-host combination (*N* = 26), of each bacterial strain is shown by color shading. **(C)** Adsorption of λ to different *E. coli* strains. Phage adsorption kinetics are shown as the average (*N* = 3) fraction of free phages remaining in solution over time and normalized with respect to the initial phage titer (100%). *Hafnia alvei* and a lysogen strain (MG1655 λ) were used as controls. The shaded area shows two theoretical adsorption curves corresponding to the first-order model (top) and the adsorption efficiency model (bottom), as described in Storms and Sauvageau (2015).

To determine the sensitivity of *E. coli* strains to different coliphages, quantitative cross-streak assays were performed (Figure 2A). None of the raw milk cheese isolates representing each SDS-PAGE cluster (Figure 2B) showed inhibition of growth when cross-streaked against four reference (λ, T4, T6, and P1) or nine somatic coliphages (Table 1) isolated from sewage, river, or seawater (Muniesa et al., 1999, 2002). Conversely, most reference *E. coli* strains showed partial or total growth inhibition. Only a uropathogenic strain (Bi), which produces a glycosaminoglycan-like capsular polysaccharide precursor to the anticoagulant pharmaceutical heparin (Cress et al., 2013), and an enteroaggregative diarrheagenic strain (W2) (Rahmouni et al., 2018) with long chain polysaccharide (Coleman et al., 1977) were resistant to all the phages. *E. coli* is a single species with strains having disparate lifestyles, from beneficial intestinal commensal to deadly pathogen (Rasko et al., 2008), as a result of divergent niche expansion millions of years ago (Hendrickson, 2009). Accordingly, the laboratory environment elicits its own ecological niche, which favors specific bacteriophage tactics to spread through laboratory-adapted strains, such as *E. coli* K-12 (Rotman et al., 2010). This variance might explain why cheese-isolated *E. coli* strains are not lysed by coliphages adapted to different host niches.

Determining the host range of a specific phage can be somewhat challenging because up to seven different host-range types, each depending on phage successfully completing different steps of the infection process, have been described (Hyman and Abedon, 2010). Although host cell killing tends to be the key determination for phage therapy, host range is often determined by success or failure of adsorption. Under laboratory conditions, bacteria evolve high levels of CRISPR-based immunity against clonal populations of phages (Common et al., 2019; Broniewski et al., 2020). However, when infected with genetically diverse phage populations, the majority of the bacterial host population evolves a surface modification preventing phage adsorption and providing generalized defense against a broader range of phage genotypes (Broniewski et al., 2020).

The interaction of λ phage with the maltose pore LamB represents the paradigm of initial phage-host binding (Chatterjee and Rothenberg, 2012) and several LamB mutations confer resistance to λ phage infection without affecting maltodextrin transport (Andrews and Fields, 2020). To investigate whether the inability of λ phage to lyse cheese strain cells was due to the lack of adsorption to the cell envelope or due to other mechanisms associated with productive growth (Maynard et al., 2010), the fraction of free λ phages was monitored for 30 min (Figure 2C). Strikingly, while adsorption of λ phage on sensitive control strains lay within the expected intervals, no decrease in the free phage titer was detected for any of the cheese-isolated strains. Additionally, the phage was expected to adsorb and inject its DNA into λ lysogen cells despite their immunity to superinfection (Fogg et al., 2010). Although these results might be explained by modifications of the receptor LamB, previous work has shown that K-12 strains with mutations for lipopolysaccharide (LPS) synthesis reduce the number of surface LamB proteins and (Randall, 1975) alter their arrangement (Yamada et al., 1981). In contrast, other studies have questioned whether the sole modification of LPS can severely impair cell infection by phage and explain the significant levels of resistance observed for deep-rough mutants (Fraser et al., 2006; Pagnout et al., 2019).

### Host Range of Coliphages Newly Isolated From Ewes

**FIGURE 3:**
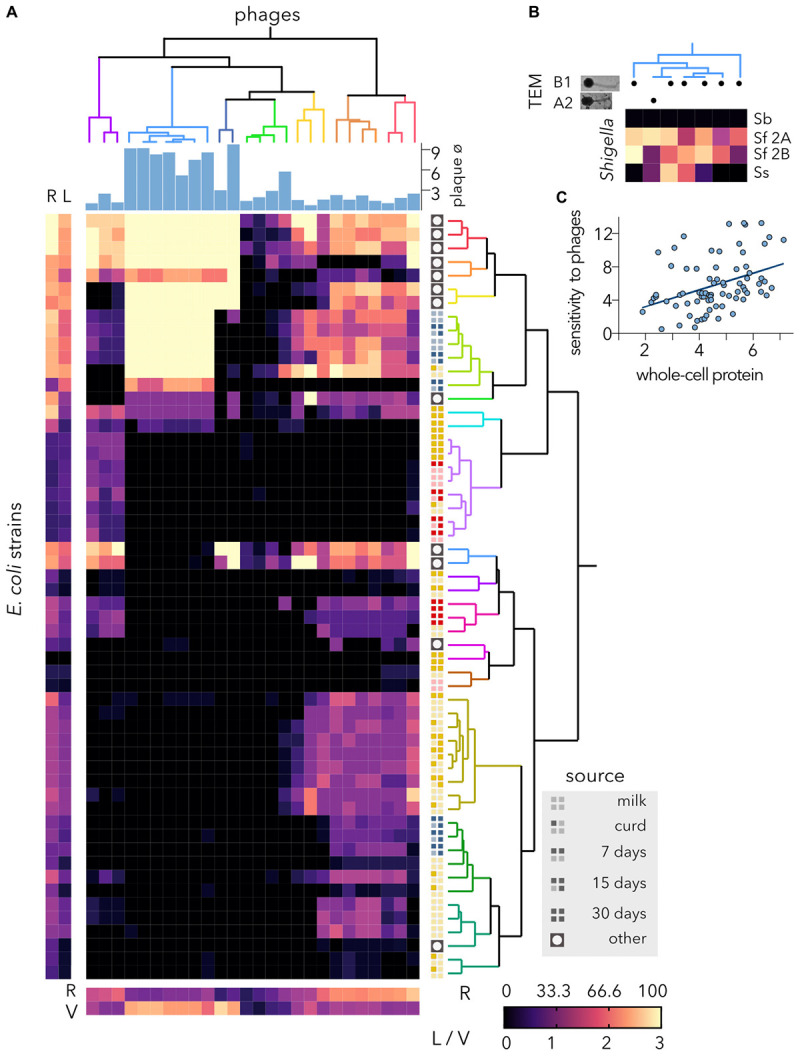
Host range of virulent isolated phages. **(A)** The heatmap represents the lysis profiles of phages versus host *E. coli* strains. Phage infection is indicated after calculating the average clearing values of several experiments (6 > *N* > 2), where clearing varies between 1 and 3; 0 = no lysis. The average values for each strain are shown on the left (bacteria) and bottom (phages). The hierarchical classifications of *Escherichia coli* strains and coliphages were performed using Ward’s method. The source of each bacterial strain is shown. The plaque diameter (mm) measured after infecting strain MG1655 is shown for each phage. R, % host range. The clearing is indicated as L (lysis) for the bacteria and V (virulence) for the phages. **(B)** Characterization of the coliphage cluster with the lowest infectivity variance. TEM, transmission electron microscopy; B1, *Siphoviridae*; A2, *Myoviridae* with elongated head. Lysis profiles of *Shigella* spp. strains. Sb, *Shigella boydii*; Sf 2A, *Shigella flexneri* 2A; Sf 2B, *Shigella flexneri* 2B; Ss, *Shigella sonnei*. **(C)** Correlation of SDS-PAGE and lysis profiles of cheese-isolated *E. coli* strains. Using the distance matrices, the correlation (*r* = 0.401) and statistical significance (*p*-value = 0.001) at an alpha of 0.05 were computed by performing a Mantel test.

To eliminate *E. coli* isolates from raw milk cheeses, ewe feces from local farms were used to isolate a total of 88 distinctive plaque-forming units. To identify the most effective and virulent phages, the plaque size and morphology were analyzed using a K-12 strain, and turbid plaque producers, which evince temperate phages, were discarded. With the remaining 26 virulent phages, a host-range matrix was built, and phages and bacteria were hierarchically clustered according to their infectivity and sensitivity profile, respectively (Figure 3A). The phages producing larger plaques on the K-12 reference strain tended to produce a more intense clearing on highly sensitive hosts, but their host range was small. Conversely, phages yielding small plaques had extended host ranges. Since it has been shown that the plaque size of lambda phage is at its maximum when the lysis time is intermediate in length and that the adsorption rate and virion size negatively impact plaque size (Gallet et al., 2011), differences in plaque size cannot directly be correlated with virulence (Abedon and Yin, 2009). One of the seven phage clusters showed low variability (see cyan cluster on Figure 3A), comprising phages with identical infectivity profile; which could indicate re-isolation of the same phage. These phages were further analyzed by TEM (Figure 3B) and one of them was member of the morphotype A2 (*Myoviridae* with elongated head), but the rest showed the same basic morphotype B1 (*Siphoviridae)* with non-contractile tails (length, ≈ 140 nm) and isometric heads (diameter, ≈ 55 nm). Considering that *Shigella* and *E. coli* K-12 have a high degree of similarity we wanted to asses whether these coliphages could propagate on some *Shigella* lineages. Although none of the phages infected *Shigella boydii*, each produced a different lysis profile on *Shigella flexneri* and *Shigella sonnei* strains (Figure 3B); thus, re-isolation of coliphages seems unlikely.

Most of the 16 resulting bacterial clusters comprised *E. coli* isolates from a single source but different ripening stages. While only one *E. coli* strain, isolated (day 30) from soft ewe cheese, was resistant to all the phages, most reference bacteria were highly sensitive to the phages and were grouped in neighboring clusters. Unlike reference and water-isolated coliphages (Figure 2B), phages isolated from feces partially inhibited the proliferation of cheese-isolated *E. coli*. Furthermore, several phages had a broad range and were able to lyse more than 50% of the bacterial hosts tested. Despite being isolated from ewe feces, the coliphages were identified using a K-12 strain adapted to laboratory conditions (Molina et al., 1998). This procedure, although fairly standard, may have favored the isolation of narrower host range phages. Nonetheless, phages able to lyse 40% of hosts are considered to exhibit a broad host range (Fong et al., 2019). Alternatively, the use of multiple host strains from different sources could have led to the isolation of broader host range phages (Ross et al., 2016).

Although correlations between the bacterial sensitivity to phages and their proteomics profile (data not shown) are not easily detectable, the high clustering of goat and semihard ewe cheese isolates in the two groups for each category (Figure 3A) suggests that there could be a correlation between the whole-protein profile and the susceptibility to phages. To evaluate this hypothesis, the Mantel test was performed using the correlation matrix between the two dissimilarity matrices (Figure 3C). To this end, only cheese-isolated *E. coli* strains were considered. The r statistic of 0.401 indicates that there is a moderate positive correlation between the matrices. The *p*-value of 0.001 indicates that these differences are statistically significant. Outer membrane proteins and LPS are typical phage receptors (Hantke, 2020), but other genes may also govern phage proliferation; thus, up to 57 *E. coli* genes when knocked out inhibit lambda phage’s ability to replicate (Maynard et al., 2010). Consequently, complex correlations between protein composition and coliphages are not unexpected.

### Analysis of Life History Tradeoffs: Correlation Between Host Range and Virulence

**FIGURE 4:**
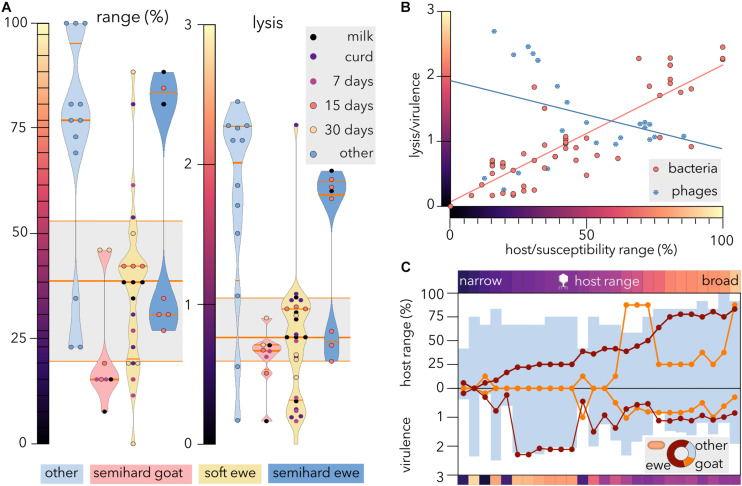
Susceptibility, range and virulence of *E. coli* and coliphages. **(A)** Violin plots represent the distributions of *E. coli* susceptibility (left) and clearance intensity (right) for different coliphages. The median and quartiles are shown as orange lines. The gray box plots show the median and quartiles of all bacterial strains. Other, reference *E. coli* strains. **(B)** Comparison of host range versus virulence (phages) and susceptibility range versus lysis (bacteria). The linear regression for bacteria (*R*^2^ = 0.72) and phages (*R*^2^ = 0.1) is shown. **(C)** Characterization of virulence and host range of the coliphages. The phages were sorted by ascending host range from left to right, and the host range and virulence were broken down into three *E. coli* categories: ewe cheese-isolated (red dots), goat cheese-isolated (orange dots), and reference (other) strains (blue bars). The color bars represent the average host range (top) and virulence (bottom) of each phage as depicted in panel **(B)**. The donut chart represents the proportion of each host category in the dataset.

To evaluate whether the origin of *E. coli* strains correlates with the susceptibility range to phages and/or with the intensity of lysis, violin plots were generated (Figure 4A) from the host-range matrix (Figure 3A). Notably, both reference strain distributions yielded median values much higher than the values of the whole dataset (range: 77 versus 38%, lysis: 2.01 versus 0.77). In contrast, *E. coli* strains isolated from goat cheese were lysed by the fewest phages (median = 15%), and the phages able to proliferate produced the minimum clearing intensity (median = 0.67). Negative effects on the physiology of *E. coli* cells might prevent the invasion of phage resistance mutations in sheep milk (Lenski, 1988). Ecological differences, such as different milk compositions (Raynal-Ljutovac et al., 2008), the cost of mutations conferring phage resistance (Sen and Nikaido, 1991), or the coevolution of coliphages and ewe isolates might explain the different susceptibility ranges in ewe and goat isolates. The microbiotas of raw milk from sheep and goats show different predominant Enterobacteria genera, with *Citrobacter* being most abundant in sheep and *Pantoea* in goats (Tabla et al., 2016, 2018). A recent comparative metagenomic analysis of fecal microbiota showed significant differences in Proteobacteria content between sheep (>26.83%) and goats (>62.03%) (Shabana et al., 2020). Neither cheesemaking technology nor stage of ripening seemed to influence the phage-sensitivity range or lysis intensity of cheese-isolated strains.

Whereas there is a high positive correlation (Spearman’s *r* = 0.83) between bacterial susceptibility range and lysis intensity (Figure 4B), the correlation between host range and virulence for the phages is negative and weaker (*r* = −0.25). Notwithstanding some phages displaying narrow host range and low virulence, virulence tended to decrease as range increased until reaching a plateau, so phages with a host range above 50% produced intermediate bacterial clearance.

For an obligately lytic phage, there is no apparent advantage in lowering virulence, as the only way to successfully infect new cells is through lysis. However, a tradeoff exists between fecundity (burst size) and latent period because release of phage offspring involves the destruction of the machinery necessary to produce more phage particles. Consequently, higher host densities select faster proliferating phages with shorter phage latent periods but smaller burst sizes (Abedon et al., 2003). Additionally, to initiate reproduction, the viral genome must be “released” from the capsid into the cell and this requirement might entail a tradeoff between stability and virulence. Accordingly, the coliphage growth rate has been previously shown to correlate negatively with host range (Keen, 2014) and with extracellular stability (De Paepe and Taddei, 2006). Overall, abundant empirical data suggest that unlike the curse of the pharaoh hypothesis, phages suffer virulence/stability tradeoffs, so stable phages with broad host ranges tend to exploit their hosts sluggishly.

To investigate the differential performance in different groups of *E. coli* strains, the coliphages were sorted by their global host range (Figure 4B), and the average range and virulence for each host group were plotted (Figure 4C). Remarkably, broad host range phages were not the most virulent for any host group. Since approximately two-thirds (64.3%) of the host dataset corresponded to ewe cheese isolates, the sorting of phages was expected to match partially with sorting by host-range values of this group. Strikingly, the average host range for reference strains never surpassed the value for ewe isolates. Although the high variability observed for goat isolates could be partially due to the smaller number of strains sampled, the differences with reference strains with a similar size might indicate diversifying selection in this particular niche. The adaptation of coliphages to withstand a particular stressor has been shown to produce negative epistatic interactions, reducing fitness when exposed to a different stressor (Heineman and Brown, 2012). These antagonistic pleiotropic effects might be explained by the small and compact genome of viruses, with some genes coding for multiple proteins that play different roles during their life cycle (Goldhill and Turner, 2014). Despite the modularity displayed for goat isolates, our results (Figures 3, 4C) reveal that ewe feces-isolated phages form a significantly nested network (*p*-value = 10^–7^) when tested against different *E. coli* strains, as evidenced by the low temperature (17.35, where 100 is the maximum value) of the host-range matrix as described elsewhere (Molina et al., in submission). To further validate these results, we conducted a meta-analysis using data from this and other 34 studies of host-phage infection assays, representing 33,428 separate attempts to infect bacterial hosts from different sources (plants, livestock, dairy, sewage, seafood, and clinical isolates) with diverse phage strains. Strikingly, only 3 (8.6%) studies statistically fit the kill-the-winner model. These findings imply gene-for-gene coevolution and would explain why the phages isolated retain the ability to propagate on host strains from different niches. Likewise, a long-term coevolution study in a natural community (Laanto et al., 2017) showed that whereas phages evolved a broader host range over time, associated with increase in genome size, the bacteria were in general resistant against phages from the past but susceptible to infection by phages from contemporary and future time points. We are currently developing (Molina et al., in submission) a new method to design phage cocktails that takes into account network structure and will allow control of *E. coli* proliferation in cheesemaking. The inclusion of bacterial lineages from diverse sources will expectedly expand its applicability.

## Conclusion

Our results are in agreement with the high bacterial diversity found in raw milk cheeses and with the high genomic diversity within the species *E. coli*, indicating that mixing multiple species of phages is required for the biocontrol of unwanted coliforms. Although broad host range phages are systematically surveyed during the formulation of phage cocktails, the evolutionary arms race with a highly versatile host such as *E. coli*, might provoke that the host range of coliphages is not necessarily a fixed property. Rather, it can show some plasticity and evolve over time. Moreover, broad host range phages face “life” history trade-offs, such as lower virulence, which might be worth considering when selecting the candidates for biocontrol. Hence, different phage cocktail formulations might be required when devising long-term and short-term biocontrol strategies.

## Data Availability Statement

The raw data supporting the conclusions of this article will be made available by the authors, without undue reservation.

## Author Contributions

FM and JR conceived and designed the experiments. AS, RT, and AG performed the experiments. FM and AS analyzed the data. RT and IR provided the bacterial strains and the cheese samples; FM wrote the manuscript. All authors read and approved the final manuscript.

## Conflict of Interest

The authors declare that the research was conducted in the absence of any commercial or financial relationships that could be construed as a potential conflict of interest.
