# Corrigendum: Diversity and Local Coadaptation of *Escherichia coli* and Coliphages From Small Ruminants

**DOI:** 10.3389/fmicb.2020.623135

**Published:** 2020-12-02

**Authors:** Felipe Molina, Alfredo Simancas, Rafael Tabla, Antonia Gómez, Isidro Roa, José Emilio Rebollo

**Affiliations:** ^1^Department of Biochemistry, Molecular Biology and Genetics, University of Extremadura, Badajoz, Spain; ^2^Dairy Department, Technological Institute of Food and Agriculture – Scientific and Technological Research Centre of Extremadura, Junta de Extremadura, Badajoz, Spain

**Keywords:** phage-host coadaptation, *Escherichia coli*, biocontrol, raw milk cheese, phage therapy, tradeoffs in life history, dairy, bacteriophages

In the original article, there was a mistake in Figure 3 as published. An outdated version of the figure was published instead of the final version. Thus, panel C was wrongly labeled as B and the actual panel B was missing. The corrected Figure 3 appears below.

**Figure 3:**
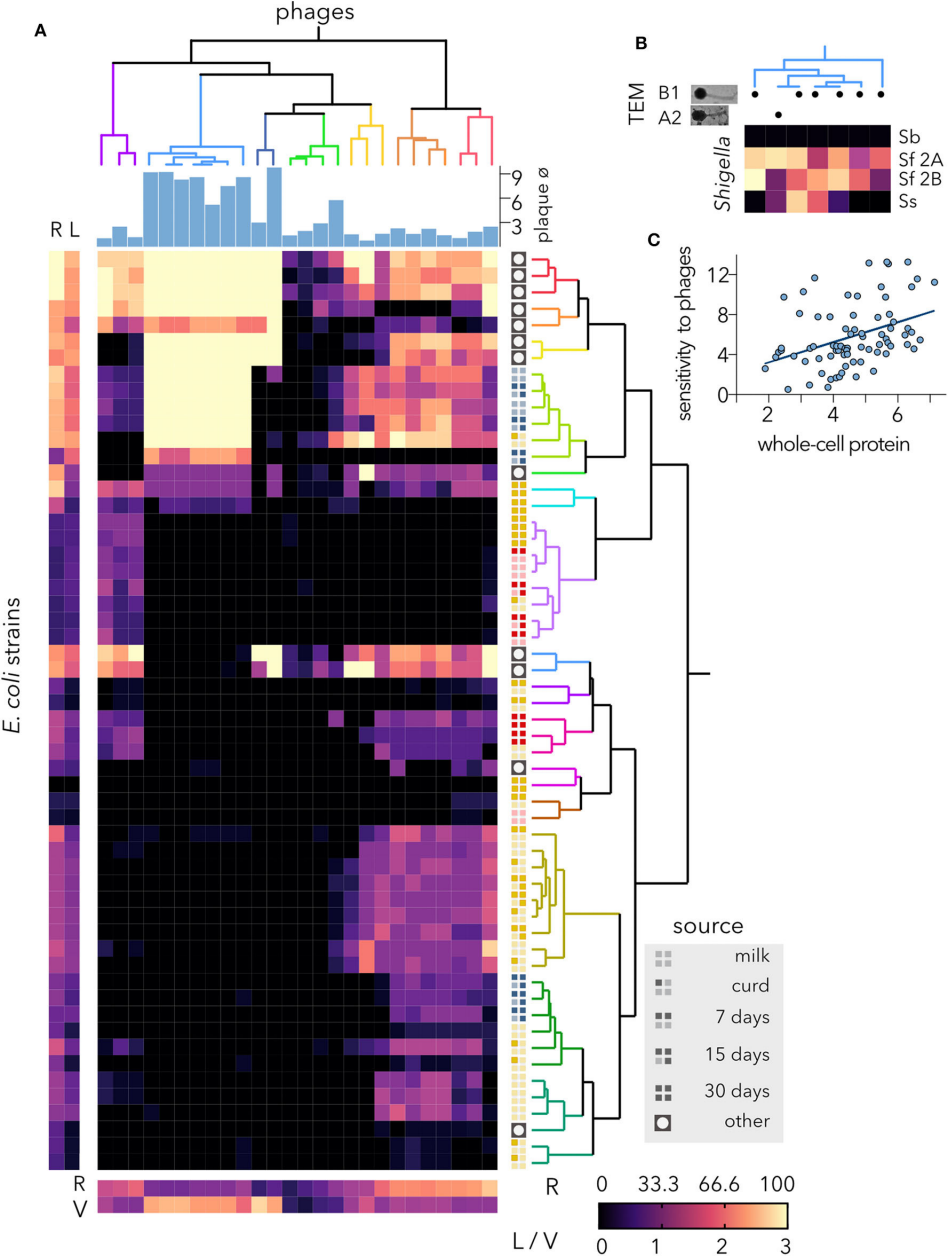
Host range of virulent isolated phages. **(A)** The heatmap represents the lysis profiles of phages versus host *E. coli* strains. Phage infection is indicated after calculating the average clearing values of several experiments (6 > *N* > 2), where clearing varies between 1 and 3; 0 = no lysis. The average values for each strain are shown on the left (bacteria) and bottom (phages). The hierarchical classifications of *Escherichia coli* strains and coliphages were performed using Ward's method. The source of each bacterial strain is shown. The plaque diameter (mm) measured after infecting strain MG1655 is shown for each phage. R, % host range. The clearing is indicated as L (lysis) for the bacteria and V (virulence) for the phages. **(B)** Characterization of the coliphage cluster with the lowest infectivity variance. TEM, transmission electron microscopy; B1, *Siphoviridae*; A2, *Myoviridae* with elongated head. Lysis profiles of *Shigella* spp. strains. Sb, *Shigella boydii*; Sf 2A, *Shigella flexneri* 2A; Sf 2B, *Shigella flexneri* 2B; Ss, *Shigella sonnei*. **(C)** Correlation of SDS-PAGE and lysis profiles of cheese-isolated *E. coli* strains. Using the distance matrices, the correlation (*r* = 0.401) and statistical significance (*p*-value = 0.001) at an alpha of 0.05 were computed by performing a Mantel test.

The authors apologize for this error and state that this does not change the scientific conclusions of the article in any way. The original article has been updated.

